# Supplementary material for: Proteogenomics and Hi-C reveal transcriptional dysregulation in high hyperdiploid childhood acute lymphoblastic leukemia
Source: Nat Commun. 2019 Apr 3;10:1519. doi: 10.1038/s41467-019-09469-3 (PMC6447538; doi:10.1038/s41467-019-09469-3)
Supplement: Supplementary file 1 — Supplementary Information [file 41467_2019_9469_MOESM1_ESM.pdf]

SUPPLEMENTARY INFORMATION FOR

Proteogenomics and Hi-C reveal transcriptional dysregulation in high hyperdiploid childhood acute lymphoblastic leukemia

Yang et al.

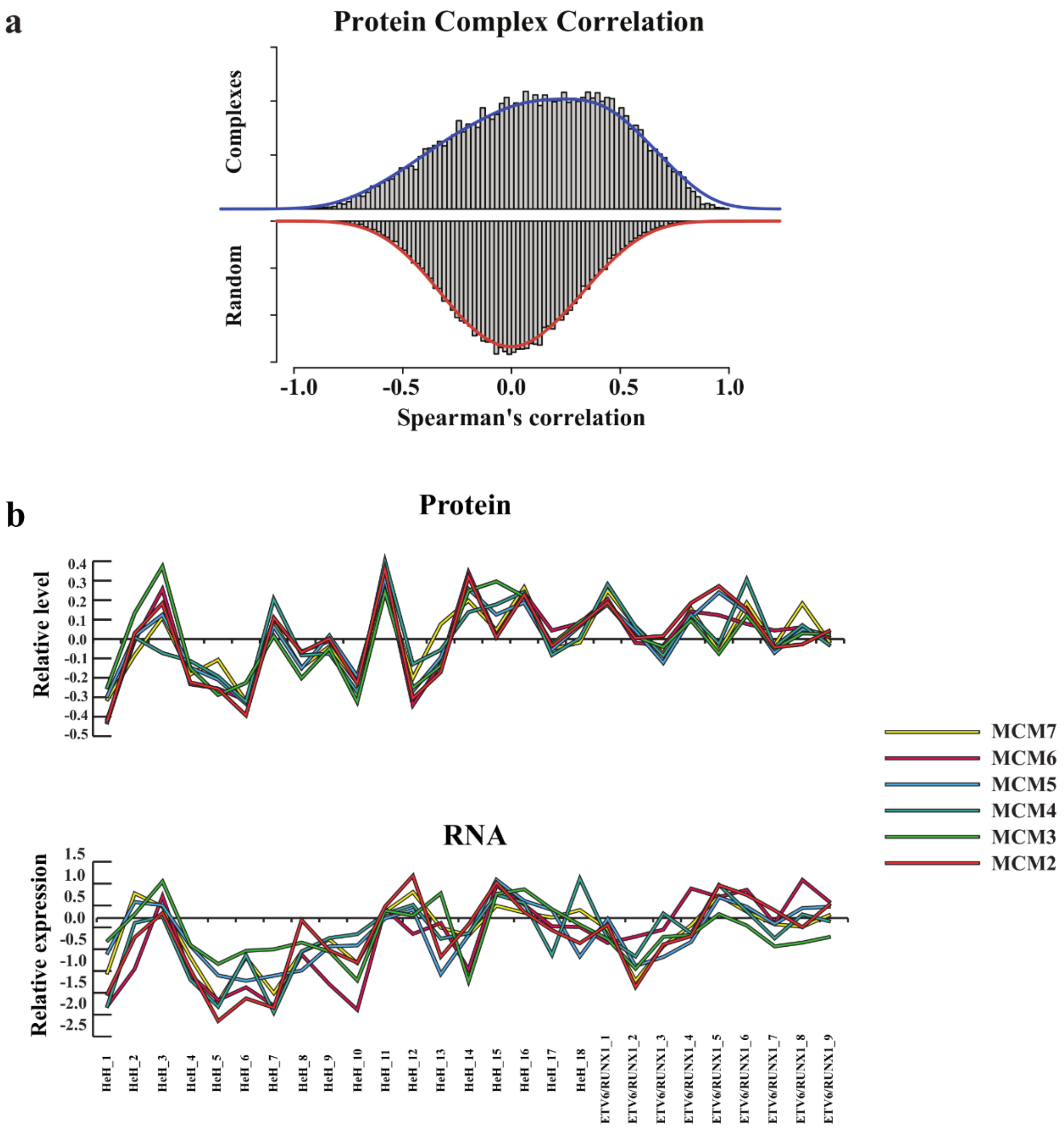

Supplementary Fig. 1. Analysis of protein complex formation a. Distribution of Spearman's correlations between protein-pairs known to form or partake in the same protein complex (CORUM) (top) compared to the distribution of Spearman's correlations of random protein pairs in the proteomics data. **b.** Example of relative levels and relative expression of members of the MCM complex in the proteomics and the RiboZero RNA-sequencing dataset, respectively.

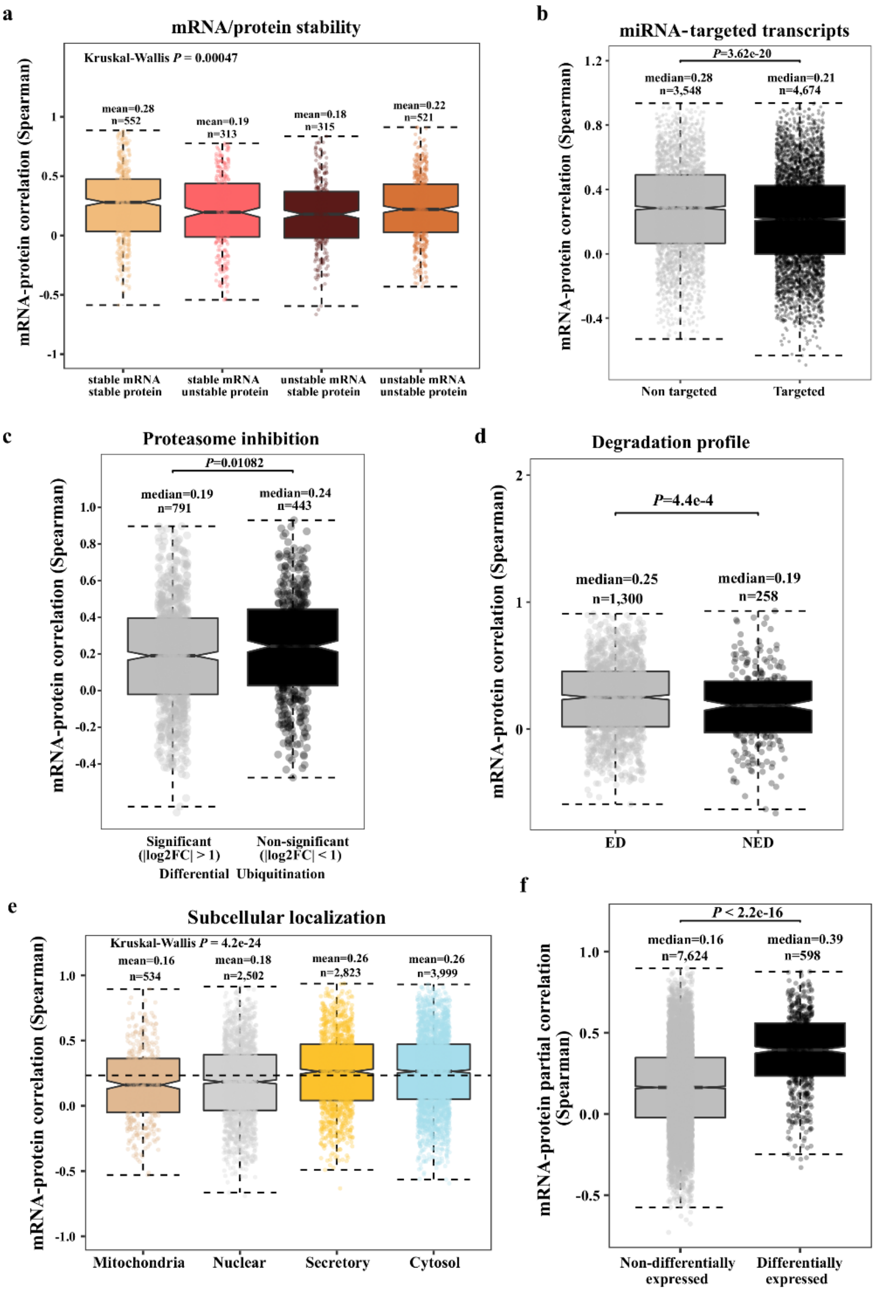

Supplementary Fig. 2. mRNA-protein correlation analysis. a. mRNA-protein correlation in relation to protein and mRNA stability, showing higher correlation for genes with similar stability on both the mRNA and protein levels. **b.** Transcripts reported to be targeted by miRNAs were compared to non-targeted transcripts, showing higher correlations for non-targeted transcripts. **c.** Association of mRNA-protein correlation to ubiquitination. mRNA-protein correlations are categorized into low (black) and high (grey) ubiquitination based on time series data following proteasomal inhibition by bortezomib. Proteins targeted by the proteasome displayed lower correlations. **d.** Comparison of proteins with an exponential (ED) and non-exponential degradation profile (NED), showing enrichment of genes with low mRNA-protein correlations among rapidly degrading proteins. **e.** Impact of protein subcellular localization on mRNA-protein correlations, showing higher correlations for secretory and cytosolic proteins. **f.** Comparison of mRNA-protein correlation distribution for differentially expressed and non-differentially expressed proteins and mRNAs. Genes that were differentially expressed between hyperdiploid and *ETV6/RUNX*1-positive leukemia displayed higher mRNA-protein correlations. For all panels, number of observations, medians, first and third quartiles, and whiskers extending to 1.5 times the interquartile range are displayed. Non-parametric Wilcoxon rank sum test (b-d, f) or Kruskal-Wallis test (a, e) was used to calculate *P*-values.

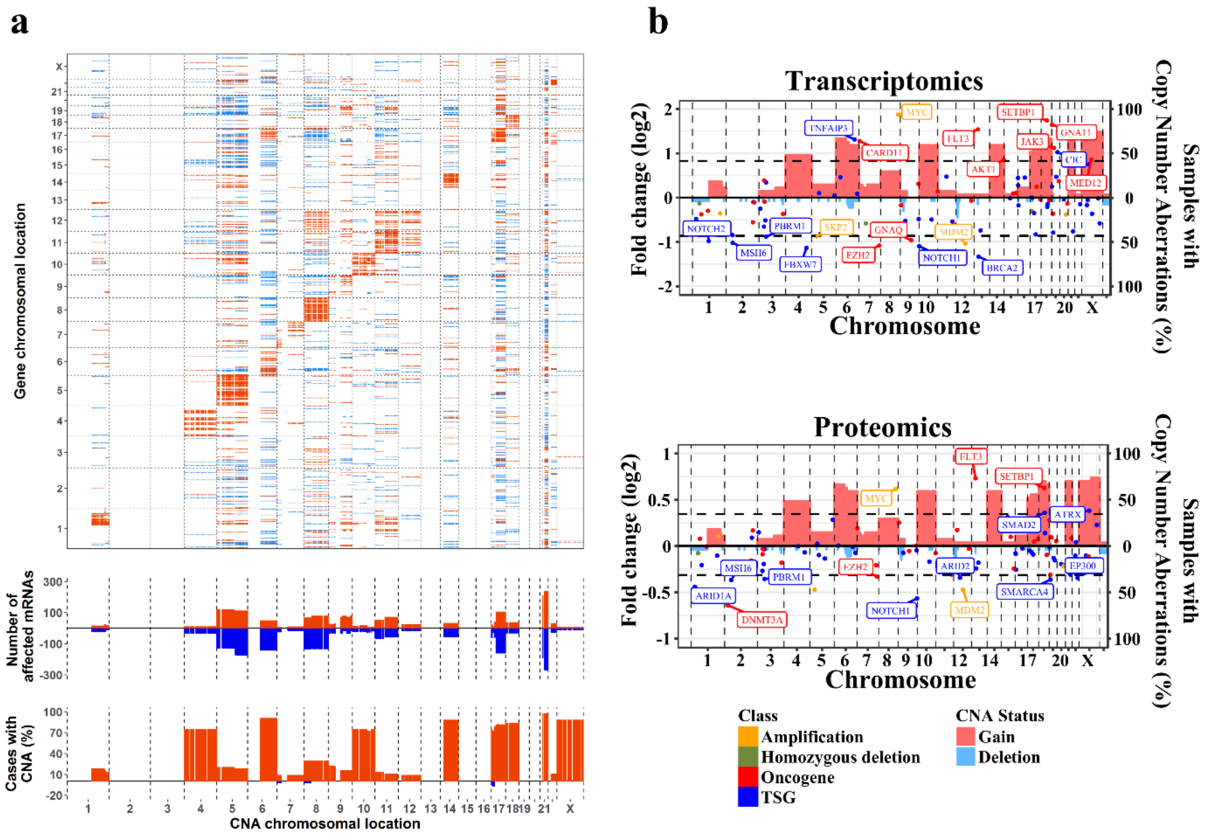

Supplementary Fig. 3. Analysis of the impact of copy number on expression in childhood acute lymphoblastic leukemia (ALL). a. Correlations of copy number aberration (CNA) (x-axes) to RNA expression levels (y-axes) based on oligo(dT) RNA-seq from high hyperdiploid cases are shown. Significant (multiple-test adjusted $P < 0.05$) positive (red) and negative (blue) correlations between CNA and mRNAs are indicated. CNA *cis* effects appear as a red diagonal line, CNA *trans* effects as vertical stripes. The fraction (%) of significant CNA *trans* effects (positive in red and negative in blue) for each CNA gene is shown. The bottom panel show the fraction (%) of leukemias harboring CNA (copy number gain in red and copy number loss in blue). **b.** Location and expression of oncogenes and tumor suppressor genes in relation to chromosomal gains in high hyperdiploid vs. *ETV6/RUNX1*-positive ALL. No association was seen between oncogenes and copy number gains or tumor suppressor genes and non-gained chromosomes.

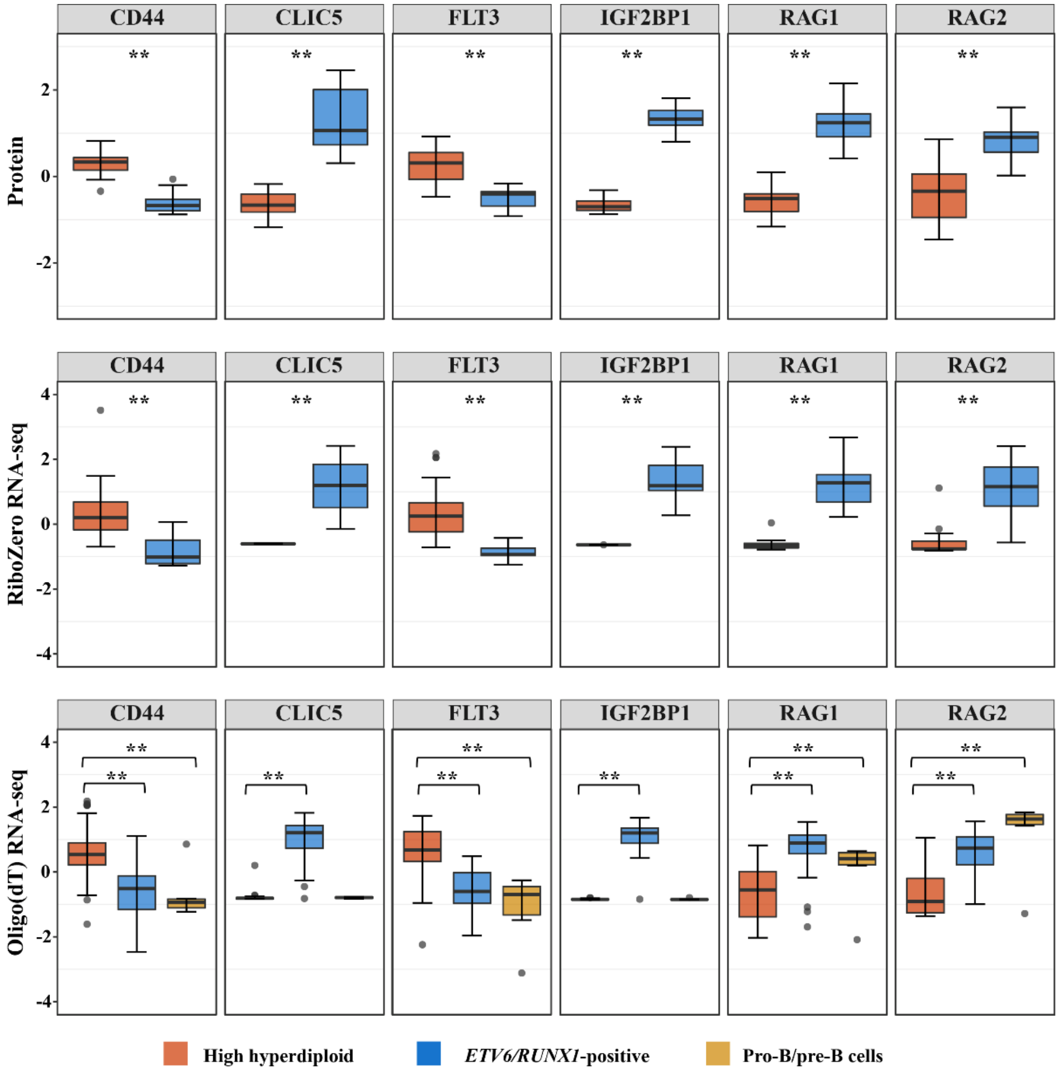

Supplementary Fig. 4. Examples of expression data from differentially expressed genes between high hyperdiploid and *ETV6*/*RUNX1*-positive acute lymphoblastic leukemia. The center of the boxplot is the median and lower/upper hinges correspond to the first/third quartiles; whiskers are 1.5 times the interquartile range and data beyond this range are plotted as individual points. Non-parametric Wilcoxon rank sum test was used to calculate *P*-values.

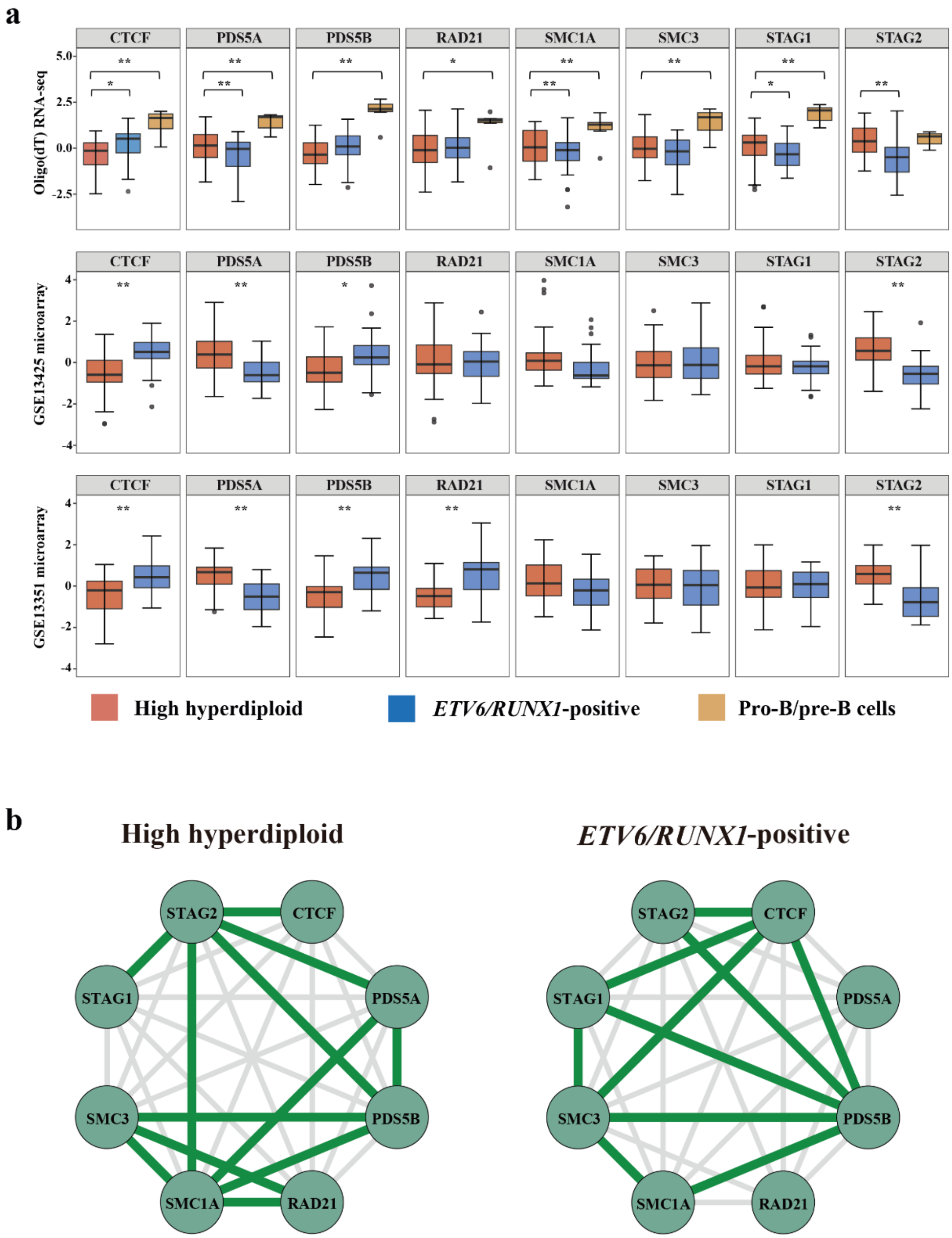

Supplementary Fig. 5. *CTCF* and members of the cohesin complex in high hyperdiploid and *ETV6/RUNX1*-positive leukemia. a. mRNA expression in acute lymphoblastic leukemia datasets. The center of the boxplot is the median and lower/upper hinges correspond to the first/third quartiles; whiskers are 1.5 times the interquartile range and data beyond this range are plotted as individual points. Non-parametric Wilcoxon rank sum test was used to calculate *P*-values. **b.** Cohesin complex members correlation. Green lines represent a Spearman's correlation coefficient of > 0.5 and grey lines are a correlation > 0 but < 0.5.

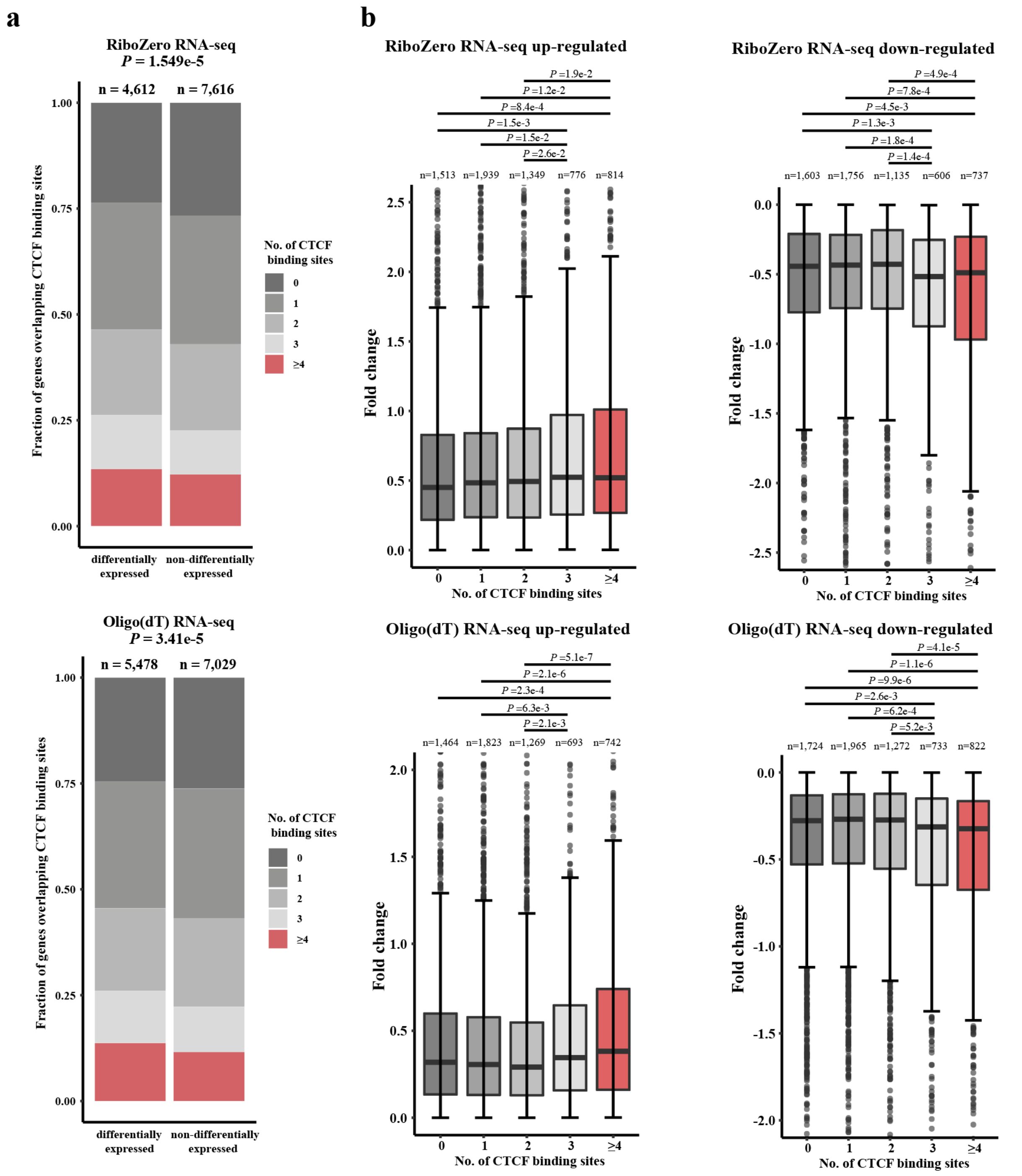

Supplementary Fig. 6. Gene expression changes and number of CTCF binding sites in high hyperdiploid leukemia. a. Genes that were differentially expressed between high hyperdiploid and *ETV6/RUNX1*-positive leukemias were strongly enriched for more CTCF binding sites in both the RiboZero (chi-squared test, *P* = 1.55e-5) and oligo(dT) (chi-squared test, *P* = 3.41e-5) RNA-seq datasets. **b.** Genes with higher numbers of CTCF binding sites in their bodies or flanking 5 kb showed significantly larger fold changes in both datasets. Two-sided Mann-Whitney U test was used to calculate *P*-values.

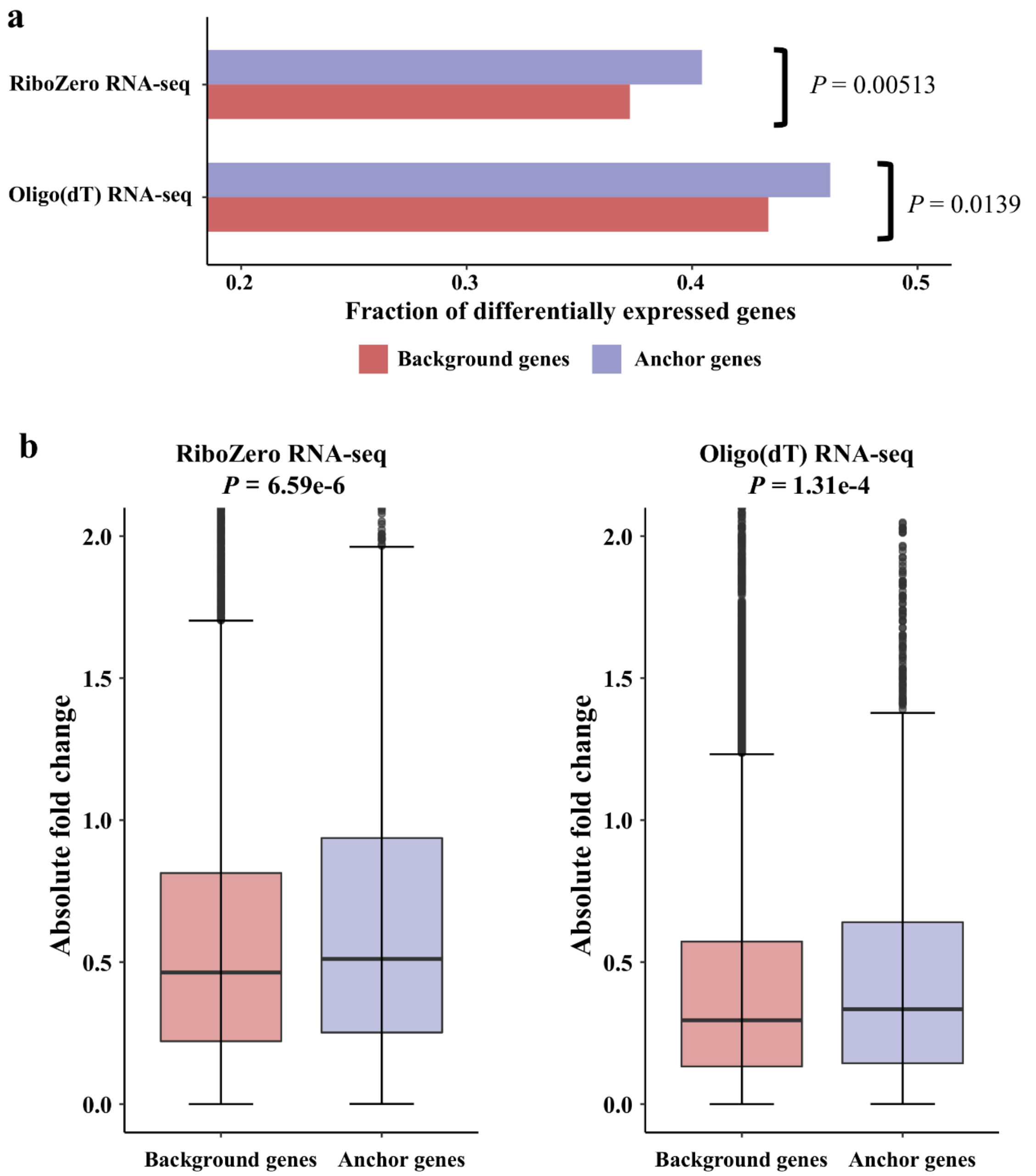

Supplementary Fig.7. Gene expression changes and CTCF/cohesin-mediated chromatin structures in high hyperdiploid leukemia. a. Fraction of anchor genes (n=1,825 and n=1,910, respectively) and background genes (n=10,403 and n=10,597, respectively) that were differentially expressed between high hyperdiploid and *ETV6/RUNX1*-positive leukemia. A significantly higher proportion of anchor genes were differentially expressed in both the RiboZero (hypergeometric test, $P = 0.00513$) and oligo(dT) (hypergeometric test, $P = 0.0139$) RNA-seq datasets. **b.** Anchor genes (n=1,825 and n=1,910, respectively) showed significantly higher absolute fold changes than background genes (n= 10,403 and n=10,597, respectively) in both the RiboZero (two-sided Mann-Whitney U test, $P = 6.59e-6$) and oligo(dT) (two-sided Mann-Whitney U test, $P = 1.31e-4$) RNA-seq datasets.

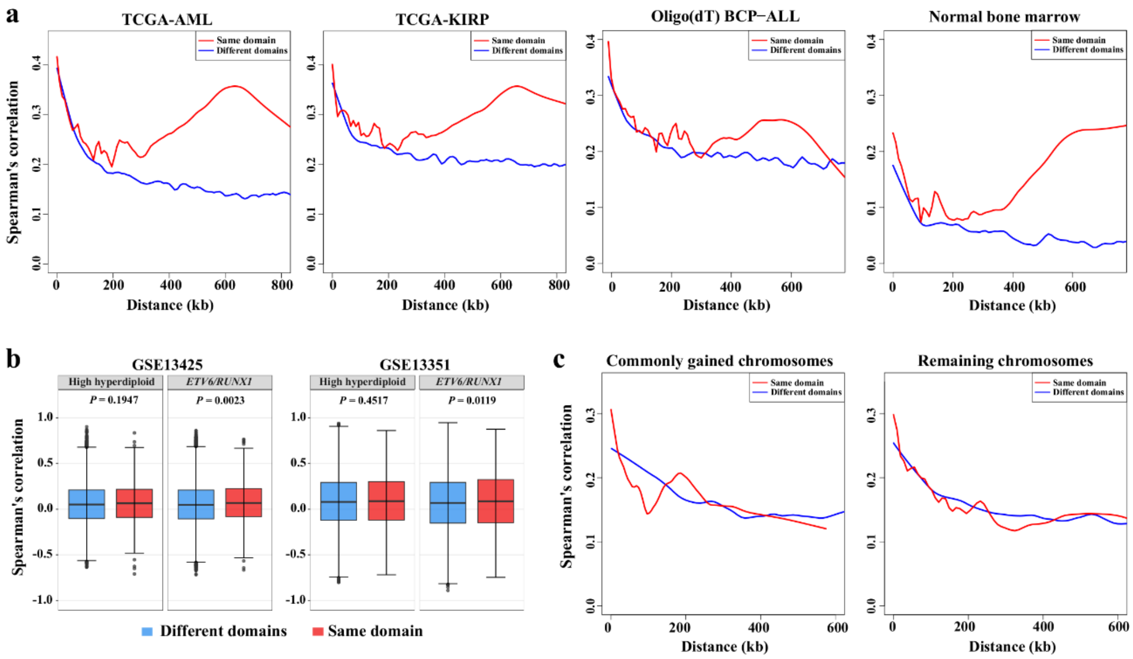

Supplementary Fig. 8. Spearman's correlation score between gene pairs as a function of distance for genes in the same or different topologically associating domains (TADs), showing higher correlation between the expression of gene pairs within the same TAD compared with gene pairs separated by a TAD boundary. a. RNA-sequencing data from acute myeloid leukemia (TCGA-LAML; n=151), papillary renal-cell carcinoma (TCGA-KIRP; n=270), childhood acute lymphoblastic leukemia (OligoT BCP-ALL; n=201), and from normal bone marrow (n=20) **b.** Microarray-based gene expression data from high hyperdiploid and *ETV6/RUNX1*-positive ALL. The center of the boxplot is the median and lower/upper hinges correspond to the first/third quartiles; whiskers are 1.5 times the interquartile range and data beyond this range are plotted as individual points. **c.** RNA-seq data from high hyperdiploid cases in the oligo(dT) dataset, analyzing commonly gained chromosomes separately from the remaining chromosomes.

Supplementary Table 1. Overview of mass-spectrometry data

	**Proteins (Gene symbol, 1% FDR)**	**Peptides (Unique, 1% FDR)**	**Peptide-spectrum matches (total)**
SET A	9,403	136,521	236,759
SET B	9,331	141,510	217,436
SET C	9,085	120,575	190,932
Total	10,138	174,966	645,127

FDR, false discovery rate
