# Supplementary material for: Proteogenomics and Hi-C reveal transcriptional dysregulation in high hyperdiploid childhood acute lymphoblastic leukemia
Source: Nat Commun. 2019 Apr 3;10:1519. doi: 10.1038/s41467-019-09469-3 (PMC6447538; doi:10.1038/s41467-019-09469-3)
Supplement: Supplementary file 3 — Description of Additional Supplementary Files [file 41467_2019_9469_MOESM3_ESM.pdf]

**Description of Additional Supplementary Files**

File Name: Supplementary Data 1
Description: Clinical and cytogenetic data from 89 cases of childhood B-cell precursor acute lymphoblastic leukemia

File Name: Supplementary Data 2
Description: Somatic mutations detected in 27 cases of childhood acute lymphoblastic leukemia

File Name: Supplementary Data 3
Description: Log2-values of relative levels of 10,138 gene-centric proteins detected and fully quantified in any one of the 3 TMT-sets

File Name: Supplementary Data 4
Description: Expression values of 8,480 proteins detected in 27 childhood acute lymphoblastic leukemias

File Name: Supplementary Data 5
Description: Expression values of 12,313 mRNAs detected in 27 childhood acute lymphoblastic leukemias analyzed by RiboZero RNA-seq

File Name: Supplementary Data 6
Description: Expression values of 12,594 mRNAs detected in 83 childhood acute lymphoblastic leukemias analyzed by oligo(dT) RNA-seq

File Name: Supplementary Data 7
Description: Enriched gene sets in high hyperdiploid vs ETV6/RUNX1-positive acute lymphoblastic leukemias based on proteomics data

File Name: Supplementary Data 8
Description: Enriched gene sets in ETV6/RUNX1-positive vs high hyperdiploid acute lymphoblastic leukemias based on proteomics data

File Name: Supplementary Data 9
Description: Enriched gene sets in high hyperdiploid vs ETV6/RUNX1-positive acute lymphoblastic leukemias based on RiboZero RNA-seq data

File Name: Supplementary Data 10
Description: Enriched gene sets in ETV6/RUNX1-positive vs high hyperdiploid acute lymphoblastic leukemias based on RiboZero RNA-seq data

File Name: Supplementary Data 11
Description: Enriched gene sets in high hyperdiploid vs ETV6/RUNX1-positive acute lymphoblastic leukemias based on oligo(dT) RNA-seq data

File Name: Supplementary Data 12
Description: Enriched gene sets in ETV6/RUNX1-positive vs high hyperdiploid acute lymphoblastic leukemias based on oligo(dT) RNA-seq data

File Name: Supplementary Data 13
Description: Hi-C sequencing data

File Name: Supplementary Data 14
Description: Topologically associated domain boundaries lost based on Hi-C

File Name: Supplementary Data 15
Description: Genes/proteins close to lost TAD boundaries that displayed significant differences in expression between high hyperdiploid and ETV6/RUNX1-positive acute lymphoblastic leukemias

File Name: Supplementary Data 16
Description: Chromosome morphology scores in childhood acute lymphoblastic leukemia
