# Supplementary material for: Proteogenomics and Hi-C reveal transcriptional dysregulation in high hyperdiploid childhood acute lymphoblastic leukemia
Source: Nat Commun. 2019 Apr 3;10:1519. doi: 10.1038/s41467-019-09469-3 (PMC6447538; doi:10.1038/s41467-019-09469-3)
Supplement: Supplementary file 20 — Reporting Summary [file 41467_2019_9469_MOESM20_ESM.pdf]

nature research

Corresponding author(s): Kajsa Paulsson

Last updated by author(s): Feb 26, 2019

Reporting Summary

Nature Research wishes to improve the reproducibility of the work that we publish. This form provides structure for consistency and transparency in reporting. For further information on Nature Research policies, see Authors & Referees and the Editorial Policy Checklist.

Statistics

For all statistical analyses, confirm that the following items are present in the figure legend, table legend, main text, or Methods section.

n/a	Confirmed	
☐	☒	The exact sample size (*n*) for each experimental group/condition, given as a discrete number and unit of measurement
☐	☒	A statement on whether measurements were taken from distinct samples or whether the same sample was measured repeatedly
☐	☒	The statistical test(s) used AND whether they are one- or two-sided<br>*Only common tests should be described solely by name; describe more complex techniques in the Methods section.*
☒	☐	A description of all covariates tested
☐	☒	A description of any assumptions or corrections, such as tests of normality and adjustment for multiple comparisons
☐	☒	A full description of the statistical parameters including central tendency (e.g. means) or other basic estimates (e.g. regression coefficient) AND variation (e.g. standard deviation) or associated estimates of uncertainty (e.g. confidence intervals)
☐	☒	For null hypothesis testing, the test statistic (e.g. *F*, *t*, *r*) with confidence intervals, effect sizes, degrees of freedom and *P* value noted<br>*Give P values as exact values whenever suitable.*
☒	☐	For Bayesian analysis, information on the choice of priors and Markov chain Monte Carlo settings
☒	☐	For hierarchical and complex designs, identification of the appropriate level for tests and full reporting of outcomes
☐	☒	Estimates of effect sizes (e.g. Cohen's *d*, Pearson's *r*), indicating how they were calculated

Our web collection on statistics for biologists contains articles on many of the points above.

Software and code

Policy information about availability of computer code

Data collection	No software was used
Data analysis	STAR v2.5.2b, HTSeq v0.9.1, Domaincaller, ProteoWizard, MSGF+ v10072, Percolator v2.08, IsobaricAnalyzer v2.0, Mutect v1.1.4, MuSE v1.0rc, BWA v0.7.17, Manta v1.3.0, Strelka v2.9.3, RUVSeq v1.14, edgeR v3.22.2, limma v3.36.1, GSEA v3.0, MatrixeQTL v2.2, HICUP v0.6.1, Juicertools v1.8.9, calCB v1.0.0, HiCNorm, InsulationScore v0.41.1, bedtools v2.27.1

For manuscripts utilizing custom algorithms or software that are central to the research but not yet described in published literature, software must be made available to editors/reviewers. We strongly encourage code deposition in a community repository (e.g. GitHub). See the Nature Research guidelines for submitting code & software for further information.

Data

Policy information about availability of data

All manuscripts must include a data availability statement. This statement should provide the following information, where applicable:

- Accession codes, unique identifiers, or web links for publicly available datasets
- A list of figures that have associated raw data
- A description of any restrictions on data availability

The mass spectrometry proteomics data have been deposited to the ProteomeXchange70 Consortium (http://proteomecentral.proteomexchange.org) with the dataset identifier PXD010175 [http://www.ebi.ac.uk/pride/archive/projects/PXD010175]. RNA-seq data have been deposited to the European Genome-phenome Archive (EGA) under the accession code EGAS00001003079 [https://www.ebi.ac.uk/ega/datasets/EGAS00001003079]. The remaining data will be available for academic research on somatic variants only by contacting the authors. Publicly available data used in this study can be found as deposited in the following datasets: Oligo(dT) RNA-seq data for ALL patients, accession number EGAD00001002112 [https://www.ebi.ac.uk/ega/datasets/EGAD00001002112]. Expression data from ALL patients, accession numbers GSE13351 [https://www.ncbi.nlm.nih.gov/geo/query/acc.cgi?acc=GSE13351] and GSE13425 [https://www.ncbi.nlm.nih.gov/geo/query/acc.cgi?acc=GSE13425]. RNA-seq dataset for AML, accession number TCGA-LAML, [https://portal.gdc.cancer.gov/projects/TCGA-LAML]. RNA-seq dataset for papillary renal cell carcinoma, accession number TCGA-KIRP, [https://portal.gdc.cancer.gov/projects/TCGA-KIRP]. Hi-C datasets for GM12878 cell line and IMR90 cell

line, accession number GSE63525 [https://www.ncbi.nlm.nih.gov/geo/query/acc.cgi?acc=GSE63525]. GM12878 CTCF ChIA-PET dataset, accession number GSM1872886 [https://www.ncbi.nlm.nih.gov/geo/query/acc.cgi?acc=GSM1872886]. GM12878 RAD21 ChIA-PET dataset, accession number GSM1436265 [https://www.ncbi.nlm.nih.gov/geo/query/acc.cgi?acc=GSM1436265].

Field-specific reporting

Please select the one below that is the best fit for your research. If you are not sure, read the appropriate sections before making your selection.

☒ Life sciences ☐ Behavioural & social sciences ☐ Ecological, evolutionary & environmental sciences

For a reference copy of the document with all sections, see nature.com/documents/nr-reporting-summary-flat.pdf

Life sciences study design

All studies must disclose on these points even when the disclosure is negative.

Sample size	No power calculation was performed. The sample size was dependent on the number of available bone marrow or peripheral blood samples and DNA and/or RNA quality measurements.
Data exclusions	No data was excluded.
Replication	For Hi-C, technical replicates were made. No other replication was done, but validation in independent datasets was performed as indicated in the text.
Randomization	Cases included in the WES/WGS, RNA-seq, LC-MS/MS and Hi-C were selected based on availability of material.
Blinding	Data analyses were performed without access to clinical information. The chromosome morphology analysis was done without knowledge of the Hi-C results. Most of the statistical analyses were performed using the nonparametric test Mann-Whitney U test (two-sided) and Spearman's rank correlation. These tests do not require the assumption of normal distribution and the values tested do not need to be categorized.

Reporting for specific materials, systems and methods

We require information from authors about some types of materials, experimental systems and methods used in many studies. Here, indicate whether each material, system or method listed is relevant to your study. If you are not sure if a list item applies to your research, read the appropriate section before selecting a response.

Materials & experimental systems

n/a	Involved in the study	
☒	☐	Antibodies
☒	☐	Eukaryotic cell lines
☒	☐	Palaeontology
☒	☐	Animals and other organisms
☐	☒	Human research participants
☒	☐	Clinical data

Methods

n/a	Involved in the study	
☒	☐	ChIP-seq
☒	☐	Flow cytometry
☒	☐	MRI-based neuroimaging

Human research participants

Policy information about studies involving human research participants

Population characteristics	Analyses were performed on 89 childhood acute lymphoblastic leukemia cases. The age of the subjects ranged from 0-16 years (median of 4 years, and mean of 5 years), and the majority of cases (52/89) were male. Detailed information for the cases can be found in Supplementary Table 1.
Recruitment	N/A
Ethics oversight	Informed consent was obtained according to the Declaration of Helsinki and the study was approved by the Ethics Committee of Lund University.

Note that full information on the approval of the study protocol must also be provided in the manuscript.
